# Controversies on Timing of Sex Assignment and Surgery in Individuals With Disorders of Sex Development: A Perspective

**DOI:** 10.3389/fped.2018.00419

**Published:** 2019-01-10

**Authors:** Tatiana Prade Hemesath, Leila Cristina Pedroso de Paula, Clarissa Gutierrez Carvalho, Julio Cesar Loguercio Leite, Guilherme Guaragna-Filho, Eduardo Corrêa Costa

**Affiliations:** ^1^PADS DSD Program, Hospital de Clínicas de Porto Alegre (HCPA), Porto Alegre, Brazil; ^2^Psycology Service, Hospital de Clínicas de Porto Alegre (HCPA), Porto Alegre, Brazil; ^3^Endocrinology Service, Hospital de Clínicas de Porto Alegre (HCPA), Porto Alegre, Brazil; ^4^Pediatrics Department, School of Medicine, Universidade Federal do Rio Grande do Sul (UFRGS), Porto Alegre, Brazil; ^5^Medical Genetics Service, School of Medicine, Universidade Federal do Rio Grande do Sul (UFRGS), Porto Alegre, Brazil; ^6^Pediatric Surgery Service, Hospital de Clínicas de Porto Alegre (HCPA), Porto Alegre, Brazil

**Keywords:** disorders of sex development, sex assignment, surgery, timing, psychosocial care, gender identity

## Abstract

Appropriate management of disorders of sex development (DSD) has been a matter of discussion since the first guidelines were published in the 1950s. In the last decade, with the advent of the 2006 consensus, the classical methods, especially regarding timing of surgery and sex of rearing, are being questioned. In our culture, parents of DSD newborns usually want their children to undergo genital surgery as soon as possible after sexual assignment, as surgery helps them to confirm the assigned sex. Developmental psychology theories back this hypothesis. They state that anatomic differences between sexes initiate the very important process of identification with the parent of the same sex. Sex-related endocrinological issues also demand early care. For example, using dihydrotestosterone cream to increase penile length or growth hormone treatment to improve final height require intervention at young ages to obtain better results. Although the timing of surgery remains controversial, recent evidence suggests that male reconstruction should be performed between 6 and 18 months of age. Feminizing surgery is still somewhat controversial. Most guidelines agree that severe virilization requires surgical intervention, while no consensus exists regarding mild cases. Our perspective is that precocious binary sex assignment and early surgery is a better management method. There is no strong evidence for delays and the consequences can be catastrophic in adulthood.

## Introduction

Disorders (or differences) of sex development (DSD) comprise a large group of conditions caused by flaws during sex determination and/or differentiation (1). The most complex clinical manifestation of DSD is genital ambiguity. It occurs primarily in the neonatal period and requires unhesitating recognition to enable planning an adequate approach.

All individuals with suspected DSD need a thorough diagnostic evaluation, including an extensive whole-body and genital physical exam, biochemical and genetic investigations, and imaging studies; the results need to be discussed by a multidisciplinary team (2). The aim is to obtain a diagnosis, at the molecular genetic level if possible, to enable prognostic predictions and genetic counseling and initiate an individualized management plan.

There is evidence that the definition of the sex of creation and acceptance of sexuality differs significantly among various societies. Therefore, when discussing sex-related issues with the family, one should not overlook social, cultural, ethnic, and religious aspects of the family or the society (3).

Efforts to standardize the management of DSD have been ongoing since the 1950s. Wilkins' work was of great importance in this aspect, as it provided for the first time, a holistic overview of DSD diagnosis and therapeutics (4, 5). A review published in 1960 was the primary document until the 2005 Chicago Meeting consensus and related publication (5, 6).

The 2006 Consensus Statement revolutionized the DSD approach. Every aspect was discussed and improved, from nomenclature to multidisciplinary care, especially psychosocial aspects (6). These recommendations were endorsed by the medical community. In 2016, the consensus update reinforced these guidelines (7).

Despite the recent advances in DSD care, several groups endorse more profound changes. In this context, we highlight a paper published last year by Viau-Colindres et al. (8). The authors advocate for a non-binary sex assignment as well as using the term intersex as a third option for birth certificates, particularly in cases of mixed gonadal dysgenesis and 46XY DSD (e.g.,: 5α-reductase deficiency and partial androgen insensitivity syndrome). They argue that as these diagnoses have higher rates of gender dissatisfaction, decisions concerning sex of rearing and urogenital surgery should be delayed to adulthood. From our point of view, this is a very dangerous proposition. First, there is a gap of almost 50 years between the Wilkins paper and the 2006 consensus. In this period, knowledge has increased, such as the first description of 5α-reductase deficiency by Imperato-McGuinley et al. (9), and the discovery of the *SRY* gene by Sinclair et al. (10). Those publications have transformed DSD management in recent years.

Another important issue is that most data concerning long-time outcomes, such as gender dysphoria in DSD (11), evaluated individuals based on old recommendations and surgical techniques, which does not necessarily reflect current contexts. This seems to be the case in our society. In Brazil, most parents demand prompt sex assignment and surgical treatment. Although our approach does not follow the current European trends, it shows very good results. Recently, De Paula et al. showed a very low sex reassignment rate in a single-center study of Brazilian patients (12). Legally the Federal Medical Council of Brazil (Resolution No. 1664 of May 2003) states in Article 2 that “patients with DSD must have ensured an early investigation conducted in a way to guarantee an adequate definition of the gender and timely treatment” (13).

Considering a bioethical point of view and the non-maleficence principle, physicians should not support decisions that they believe are harmful to the child. In these cases, one might argue that irreversible surgical feminization or masculinization can harm the adult that the child will become. Yet the option of inaction is harmful, as it commits the patient to a childhood of ambiguous genital anatomy; this may be particularly difficult if the parents' and society's values do not support it. Each option has its advantages and disadvantages. The principle of autonomy and respect supports preserving options for children until they can decide for themselves; however, this is a social experiment with consequences still unknown (14).

Therefore, it seems very premature to advocate for a radical change in concepts. In our experience, prompt management is still the better choice for a newborn with ambiguous genitalia. There are some relevant endocrinological, surgical, and psychological aspects that reinforce our position.

## Endocrinological Aspects

Some endocrinological issues should be considered when determining the best surgical time. For example, when the sex is clearly, with a micropenis, early administration of testosterone or dihydrotestosterone cream to increase penile length can be effective. Androgen receptors decrease during adolescence and early adulthood; therefore early use of testosterone allows a better response (15, 16). Sometimes, depending on urethral malformation or ventral curvature, this treatment would be difficult to implement before surgery without discomfort.

Another important issue is height. Women with complete androgen insensitivity syndrome are reportedly as tall as males (174 cm) and that chromosomal DSD adults are as tall as or shorter than females (17). This difference in height may be linked to SHOX (*short stature homeobox gene*) haploinsufficiency (18). We know that adult height follows a child's biological sex and not the sex of rearing. This has enabled growth hormone (GH) treatment for Turner syndrome patients (19). The use of somatropin for DSD patients is also described (20). However, if the child's predicted final height is unfavorable and binary sex assignment is delayed until adulthood, the individual could miss the opportunity for GH treatment if they decide on a male sex of rearing.

## Surgical Aspects

During the 2016 consensus update, 32 expert surgeons were consulted and there was no consensus regarding the indications, timing, procedures, and developments in DSD surgical outcomes (7). However, some groups reported results that justify the early approach advocated by our group. Nevertheless, some points obtained universal consensus, most importantly, the need for a multidisciplinary management team and accurate follow-ups.

### Masculinizing Genitoplasty

When the male sex is assigned, the recommended management shows no major variations. Surgical timing varies somewhat, with the ideal period being between 6 months and 2 years of age (21) or as recommended by the American Academy of Pediatrics (AAP), with tolerance up to 18 months (22). More complications are reported in surgeries performed on boys after 1 year of age and adolescents (22). The surgical technique also did not vary much; surgeons mostly (49–60% of procedures) preferred to use staged techniques to correct proximal hypospadias (21, 23).

### Feminizing Genitoplasty

The biggest concern is regarding the ideal time for genitoplasty in girls. Most literature recommends that surgery be performed during the first year of life (24, 25). It can be performed as a staged procedure, reducing the clitoromegaly and external genitalia reconstruction (25), but this practice can lead to disposal of tissue that may be important in future vaginal reconstruction (22). The most relevant arguments for early surgery are better quality of the genital tissues, better vascularization secondary to postnatal maternal estrogens, and possible reduction of parental and child anxiety regarding the appearance of the external genitalia (25, 26). However, vaginal dilation is not recommended in childhood (27).

Milhada et al. compared two cohorts, from 2001 and 2012 (one before and one after the 2006 consensus). The last cohort showed an increase in the overall postoperative cosmetic result, where 76% were considered good or satisfactory, compared to 54% in 2001 (*p* = 0.021); the need for major vaginal surgery reoperation decreased, from 75% in 2001 to 24% in 2012 (*p* = 0.001) (28). Crawford et al. reported similar data among girls, with good (85%) and satisfactory (15%) anatomical/cosmetic results (29).

Jesus (30) reported that most patients report good clitoral sensation after feminizing genitoplasty; complaints of poor clitoral sensation were directly related to multiple surgeries, clitoral amputation, or complete atrophy or non-preservation of the neurovascular bundle. Most patients (70.8%) considered the timing of their surgery correct (mean age at first surgery 2.1 years), while 3 patients said they were operated too late (9, 14, and 17 years) (30). Ellens et al. reported that mental health problems and depressive symptoms in parents of children with DSD improved progressively 6 to 12 months after surgery (31).

### Gonads

There are no major variations in the approach to the gonads. The removal of confirmed streak gonads is indicated if Y material is present (22, 27). In patients with complete androgen insensitivity syndrome (CAIS), conservative management of gonads is suggested until puberty, as there is a small risk of malignancy (27). Furthermore, in patients assigned as boys, the time for orchidopexy has decreased significantly after the 2006 consensus (from 54.5 ± 43.0 months to 41.4 ± 45.3 months) (32).

## Psychological Aspects

The absence of an initial definition of the child's sex can cause an emotional impact in the parents (33). The social environment, depending on the current culture, may not tolerate “rare” conditions, and lack of knowledge about DSD in the lay public provokes prejudiced reactions against the child and family. Parents often get divorced because DSD in child becomes too difficult to discuss, creating a rift between the couple (34).

Sexual assignment followed by hormone therapy and surgery alone do not solve the issue, as there is still no clear understanding regarding the psychological adaptation of the individual to the designated sex. This is because the sex assigned at birth will only be validated by a set of organic and psychological characteristics. For example, the sex of rearing used by parents to educate the child and the identity and gender role, which results from different levels of sexual distinction: genetics, nuclear, gonadal, phenotypic, and psychosocial (35).

It is important to discuss the psychosocial construction of the DSD patient, since this is what determines the adequacy between sexual identity and gender identity. A better fit to the assigned sex indicates less conflict with the genital anatomy and development of a consonant gender identity. The parents' role in this construction is critical, as they are responsible for the child's first relationships with the outside world, projecting his representations about his sexual identity, and the subsequent relationship between this and gender identity (33).

Stoller (36) expands some conceptual definitions regarding sex and gender based on Freud's theories of psychosexual development. The author proposes a distinction between sex and gender, relating the former to the biological condition of being male or female and the latter to the development of the behavioral and characterological characteristics related to this condition (masculinity and femininity) (36). Masculinity or femininity is defined by (36) as any quality that is felt by those who possess it as male or female (36). Thus, masculinity or femininity is an algebraic sum of a dense mass of beliefs that make the individual feel that they qualitatively belong to either sex. Besides the biological basis, the person obtains these convictions from parental attitudes, primarily in childhood, as these attitudes are fairly similar to those held by society. Therefore, cultural markers that give an individual the notion that he is male or female are fundamental, but changeable according to prevailing cultural patterns (36).

Money et al. investigated children with DSD and proved that the landmark gender identity formation occurs between the ages of 18 and 36 months (37). It was observed that, despite their undifferentiated sexual anatomy, children with DSD could develop a stable identity if raised unambiguously as members of either sex (37), meaning if parents give these children an education within normal limits, like any other child born without DSD.

Currently, there is a significant discussion regarding the rights of patients born with DSD who undergo genital surgeries early in life. The primary fear of those who manage such cases is that the originally designated sex will later be repudiated by the patient. However, the methodological problems implicit in longitudinal research on the psychological development of individuals with DSD are also under discussion (38). A major obstacle in this discussion is the heterogeneity of the cases. The important question is whether adult patients dissatisfied with management performed more than 30 years prior should regulate the treatment of patients born with DSD in the present. The conceptual framework and scientific knowledge about the psychology of child development should help in this debate (33, 39, 40).

Meyer-Bahlburg (41), Slijper et al. (42), and Zucker (43) reported that the perception of atypical genitalia causes negative body perception, leads to confusion in relation to the sense of masculinity and femininity, and can therefore lead to behaviors inconsistent with those expected for the sex defined at birth. Thus, genital surgery performed after DSD investigation, is a preventive intervention, reinforcing the child's belonging to a defined sexual identity. This is considered the foundation on which the future gender identity of the child is based. In addition, sexual identity is understood as one of the elements that form an individual's subjective identity, enabling the formation of a wide range of personality characteristics. Thus, the anatomical criterion used to define the sexual identity of the newborn is the point where this aspect of the individual begins, which finds in the birth registry a kind of validation of the “true sex” to which the subject belongs.

According to Santos (35), gender presupposes a social and historical construction that depends on experiences and subjective experiences that belong to a group, race, ethnicity, and social class. Gender stands out in social relations as the organizer of social and sexual identities.

The concept of gender was incorporated by sociology, according to Louro (44), as a reference to the social organization of the relationship between the sexes. It was part of the Brazilian academy in a space dispute with women's studies, emerging from feminist movements after the 1960s. Development of this concept is strongly influenced by different areas, such as linguistics, psychoanalysis, psychology, anthropology, and history, all of which are responsible for investigating and demonstrating the cultural variability of behaviors, acquisitions, and skills considered to be male and female. The studies produced by these different areas develop a perspective in which masculinity and femininity are fundamentally constructed and represented by culture. It is the beginning of a phase of social determinism, which has been developing in parallel with advances in medical technology, which in itself intrinsically brings biological determinism.

Lately, political movements and gender activities have gained momentum worldwide, demanding fluidity about gender identity. This phenomenon seeks to include patients born with DSD, understood by social movements as subjugating the medical hegemony and the “necessity” of hormonal and surgical treatments. However, political movements often do not consider that affected patients and their families seek treatment because they think it is necessary to improve their quality of life. Most patients who seek reference centers later in life, either because of late diagnosis or being lost to follow-up, wish to undergo genital correction surgery. Usually, women desire more feminine bodies and men desire development of their sexual characteristics (45, 46). Most patients wish for “normal” lives and fear social rejection (47).

Freud (48), in his studies on the sexual development of children, has always been concerned with the origins and maintenance of masculinity and femininity as essential aspects of psychoanalytic theory. Although Freud did not refer specifically to gender identity, in describing and conceptualizing the identificatory processes, he brought important elements to understand a human's identification with the male or female sex. In this sense, identification with biological sex implies a conflictive process originated by the opposition of what is male and female. This process, through which every human being begins to assume his or her own sex, indicates the male and female sexual dispositions inherent in every person. The resolution of conflicts and the establishment of identification with the parental figures are important aspects of the constitution of the subject's nuclear gender identity. Thus, the attitudes of parents in the identification process through which the whole child passes are important, and identification with one of the parental figures is essential for the adequate development of gender identity.

Hemesath (33) demonstrated in her study that for the parents of a newborn with DSD, the appearance of the child's genitalia is the fundamental factor that marks the child's sexual identity, on which the gender identity is ultimately constructed. In addition to the child's atypical genitalia at birth, a consensus was found among the parents participating in the mentioned study that it is impossible to develop a healthy gender identity without the definition of a prior sexual identity. In fact, an individual's subjective identity is organized based on the sexual identity. Thus, parents of children with DSD understand that the child's gender identity is built based on 3 criteria: 1—defined genital anatomy (i.e., after surgical correction); 2—breeding sex they employ with the child throughout their lives; and 3—social recognition that the child will have at later stages of development.

## Conclusions

In conclusion, there is a lack of data to support the delay the sex assignment (as well as a non-binary assignment) and consequently, genital surgery. Based on this and on many years' experience with such patients and their parents, we recommend early surgery, because re-adaptation of the baby's genitalia, according to the sexual designation it receives, generates relief in the parents and triggers the construction of the gender of the individual with DSD.

In this study, the authors attach great importance to studies with specific delimitations in each culture, as a way to understand the psychological and emotional phenomena with this bias. Differences in parental educational strategies between different countries are already recognized through various studies. In our population this is evident, and it marks the development of patients with DSD.

## Author Contributions

TH and EC: contributed to design of the study, wrote and reviewed the manuscript and answered to reviewers. LP, JL, CC, and GF: contributed to design, and wrote and reviewed the manuscript.

### Conflict of Interest Statement

The authors declare that the research was conducted in the absence of any commercial or financial relationships that could be construed as a potential conflict of interest.
